# Calcium Homeostasis and Muscle Energy Metabolism Are Modified in HspB1-Null Mice

**DOI:** 10.3390/proteomes4020017

**Published:** 2016-05-04

**Authors:** Brigitte Picard, Malek Kammoun, Mohammed Gagaoua, Christiane Barboiron, Bruno Meunier, Christophe Chambon, Isabelle Cassar-Malek

**Affiliations:** 1 International Institute of Agronomical Research (INRA)-Vetagro Sup, UMR1213, Saint-Genès-Champanelle F-63122, France; malek.kammoun@utc.fr (M.K.); gmber2001@yahoo.fr (M.G.); christiane.barboiron@clermont.inra.fr (C.B.); bruno.meunier@clermont.inra.fr (B.M.); isabelle.cassar-malek@clermont.inra.fr (I.C.-M.); 2 INRA-Metabolism Exploration Plateform (PFEM), Saint-Genès-Champanelle F-63122, France; Christophe.chambon@clermont.inra.fr

**Keywords:** 2D-electrophoresis, MS-MS, skeletal muscle, *HspB1*-null mouse

## Abstract

Hsp27—encoded by *HspB1—*is a member of the small heat shock proteins (sHsp, 12–43 kDa (kilodalton)) family. This protein is constitutively present in a wide variety of tissues and in many cell lines. The abundance of Hsp27 is highest in skeletal muscle, indicating a crucial role for muscle physiology. The protein identified as a beef tenderness biomarker was found at a crucial hub in a functional network involved in beef tenderness. The aim of this study was to analyze the proteins impacted by the targeted invalidation of *HspB1* in the *Tibialis anterior* muscle of the mouse*.* Comparative proteomics using two-dimensional gel electrophoresis revealed 22 spots that were differentially abundant between *HspB1*-null mice and their controls that could be identified by mass spectrometry. Eighteen spots were more abundant in the muscle of the mutant mice, and four were less abundant. The proteins impacted by the absence of Hsp27 belonged mainly to calcium homeostasis (Srl and Calsq1), contraction (TnnT3), energy metabolism (Tpi1, Mdh1, PdhB, Ckm, Pygm, ApoA1) and the Hsp proteins family (HspA9). These data suggest a crucial role for these proteins in meat tenderization. The information gained by this study could also be helpful to predict the side effects of Hsp27 depletion in muscle development and pathologies linked to small Hsps.

## 1. Introduction

Among beef sensory qualities, tenderness is the most important criteria because it is characterized by a high and uncontrolled variability, which is one reason for consumer dissatisfaction as beef consumers seek meat of high and consistent quality [1]. Therefore, beef tenderness has been the subject of a lot of studies over the past years. Moreover, today beef tenderness can only be determined after slaughter at the time of eating. Thus, the beef sector is looking for biological or molecular indicators which could be used in prediction tools to classify carcasses at slaughter according to their tenderness, or to predict the ability of live animals to produce good meat with controlled tenderness. To achieve this goal, several genomic programs combining genetics, transcriptomics, proteomics and biochemistry have been carried out over the past years (for review: [2]). This has made it possible to highlight a number of protein biomarkers of tenderness [3]. These biomarkers were analyzed using bioinformatics in order to establish a functional network involved in beef tenderness [4]. This network was constituted of 330 proteins involved in different biological functions such as muscle structure, metabolism, contraction, oxidative stress and apoptosis. In this analysis, the gene ontology (GO) functions “apoptosis” and “regulation of apoptosis” represented 16% of the overall occurrences of the interactome GO functions, demonstrating the importance of apoptosis (cell death program) in the determinism of tenderness. This is in accordance with the theory of Ouali *et al.* [5], that apoptosis would initiate cellular proteolysis during the early stage of tenderization, just after bleeding. This mechanism would be associated with the destruction of the cytoskeleton under the action of proteases such as caspases.

In the interactome related to tenderness [4], heat shock proteins (Hsps) were highly represented. In particular, Hsp27 encoded by the *HspB1* gene was a hub protein, suggesting its key role in tenderness. In order to depict the molecular mechanisms involved in tenderness through the Hsp27 protein, we engineered a knock-out mouse by targeted invalidation of the *HspB1* gene [6]. In an initial study, we used a network-basis approach combined to biochemistry to reveal Hsp27 interactors that may be related to tenderness. However this approach enabled predicting Hsp27 targets solely in the *Soleus* muscle [6]. In another study, we also provided evidence that the *HspB1*-null mouse displays ultrastructural abnormalities in the myofibrillar structure of mutant mice [7]. Thus, Hsp27 could directly impact the organization of muscle cytoskeleton and therefore have implications in tenderness. The aim of the present study was to understand the biological mechanisms involved in beef tenderness through the implication of Hsp27. We used a model of a knock-out mouse to propose some hypotheses which will be validated in cattle in future experiments. We used proteomics (two-dimensional gel electrophoresis and mass spectrometry) to reveal without *a priori* the proteins whose abundances are modified in the m. *Tibialis* of *HspB1*-null mouse. A bioinformatic analysis was performed to identify pathways likely involved in the functions of Hsp27 which could be important for beef tenderness.

## 2. Experimental Section

### 2.1. Animals and Experimental Procedure

Constitutive knock-out mice with a gene deletion of *HspB1* (*HspB1*-null mice) were obtained through targeted insertion (homologous recombination). A portion of the 1381 bases of the genomic DNA, including the three exons of the *HspB1* gene, were replaced with a neomycin resistance gene and *LacZ* reporter gene (Mouse Micer vector set 369N20) [8]. The mice were housed at the experimental plant of nutrition and microbiology of the National Institute for Agricultural Research (INRA-France), in a temperature and humidity controlled room under a 12-hour light and dark cycle. They were fed *ad libitum*. The experimental procedures and animal holding respected French animal protection legislation, including the licensing of experimenters. They were controlled and approved by the French Veterinary Services and the “Comité Régional d’Ethique en Matière d’Expérimentation Animale Région Auvergne” ethics committee (agreement number CE 84-12). All efforts were made to minimize suffering.

### 2.2. Muscle Samples

The *HspB1*-null mice and their control littermates were sacrificed at 12 weeks postnatal. Samples of the *Tibialis anterior* muscle (fast glycolytic) were taken immediately after sacrifice, frozen in liquid nitrogen and kept at −80 °C until protein extraction. Total protein extractions were performed on five mice from each genotype according to Bouley *et al.* [9] in a denaturation/extraction buffer (8.3 M urea, 2 M thiourea, 1% Dithiothreitol (DTT), 2% CHAPS) and stored at −20 °C until use. This buffer does not allow for a good solubilization of proteins from the extracellular matrix, which had not been considered in our study. The protein concentration was determined by spectrophotometry using the Bradford assay [10]. The extraction yield was the same for the two genotypes.

### 2.3. Proteomic Analysis

#### 2.3.1. Two-Dimensional Gel Electrophoresis

Isoelectric focusing (IEF) was carried out in a PROTEAN IEF-cell (Bio-rad, Hercules, CA, USA) with 18 cm-long IPG (immobilized pH gradient) strips with a pH range of 5–8. The method used was previously described by Bouley *et al.* [9], with the exception that, after a desalting step (50 V, 7 h), the proteins were separated according to the following conditions: 200 V for 1 h; 500 V for 1 h; 1000 V for 3 h, increasing to 8000 V over 9 h; 8000 V continuously until 73,500 Vh.

Sodium dodecyl sulfate polyacrylamide gel electrophoresis (SDS-PAGE) (T = 11%, C = 2.6%) was run on a PROTEAN plus Dodeca cell system (Bio-Rad, Hercules, CA, USA) at 40 V for 1 h, with 15 mA per gel and 110 V until the Bromophenol blue migration front reached the bottom of all the gels.

Three replicates of two-DE (Dimensional electrophoresis) gels of HspB1-null mice (*n* = 5 samples) and their control littermates (*n* = 5 samples) were carried out. The two-DE gels were stained with G250 Colloidal Coomassie Blue for 72 h, as described previously [9]. This dye, which is known to be less sensitive than fluorescent dyes [11], has been chosen for technical and economic reasons and also for its usefulness for protein identification by mass spectrometry. After staining, gels were destained until sufficient background was cleared before digitization. Two-DE gel images were acquired using an Expression 10000XL Pro Scanner (Epson, Nagano, Japan) at 300 dots per inch (dpi). Image warping, spot detection and volume quantification were carried out using the SameSpots v3 software (Nonlinear Dynamics, Newcastle, UK).

#### 2.3.2. Protein Identification by Mass Spectrometry LC-MS/MS

Stained spots of interest were excised by hand from at least three different replicate gels for each of the five samples for each genotype and subjected to the following treatments. First, the spots were washed in 25 mM ammonium bicarbonate −5% acetonitrile for 30 min and twice in 25 mM ammonium bicarbonate −50% acetonitrile for 30 min each. The spots were then dehydrated with 100% acetonitrile. The dried gels were reswelled in 25 mM ammonium bicarbonate and digested at 37 °C for 5 h with 10 to 15 μL (depending on the gel volume to be treated) of trypsin solution (12 ng/μL; V5111, Promega, Madison, WI, USA). Following peptide extraction with 8 to 12 μL of acetonitrile and concentration, the peptide mixtures were analyzed by online nanoflow liquid chromatography using the Ultimate 3000 RSLC (Dionex, Voisins le Bretonneux, France) with nanocapillary columns of 15 cm length × 75 μm I.D. (Acclaim Pep Map RSLC, Dionex, Sunnyvale, CA, USA). The solvent gradient increased linearly from 4% to 50% Acetonitrile (ACN) in 0.5% formic acid at a flow rate of 300 nL/min for 30 min. The elute was then electrosprayed in a LTQ-VELOS mass spectrometer (Thermo Fisher Scientific, Courtaboeuf, France) through a nanoelectrospray ion source which was operated in a CID top 10 mode (*i.e.*, one full scan MS and the 10 major peaks in the full scan were selected for MS/MS). The Thermo Proteome Discoverer v1.2 (Thermo Scientific, Waltham, MA, USA) was used for raw data file processing and Mascot (V2.2, www.matrixscience.com) for protein identification. For the mouse database searches (http://www.uniprot.org, 50287 seq), the following parameters were considered: peptide mass tolerance was set to 1.5 Da, fragment mass tolerance was set to 0.8 Da and a maximum of two missed cleavages were allowed. The variable modifications were methionine oxidation (M) and carbamidomethylation (C) of cysteine. A protein was considered valid when a minimum of three unique peptides originating from one protein were shown to be statistically significant (individual peptide score >39; *p* < 0.05).

Protein spots with more than one protein identified in the mixture were considered for biological interpretation only if they passed both of the following criteria: (i) the relative abundance based on the number of sequenced peptides per protein (EmPAI protein content (mol%) (http://www.matrixscience.com/help/quant_empai_help.html)) for the most abundant protein was larger than 50%; and, moreover; (ii) the ratio of the EmPAI for this most abundant protein to EmPAI for the second most abundant protein in the spot mixture was at least 2).

#### 2.3.3. Western-Blotting Validation

The variation in protein abundance between the two genotypes was validated using a quantitative Western blotting technique. It was processed exactly as described in Chaze *et al.* [12]. Fifteen or 30 μg of proteins were separated by gel electrophoresis using 1D SDS-PAGE for 2 h according to the Laemmli method [13]. After migration, the proteins were transferred onto Polyvinylidene fluoride (PVDF) transfer membrane Millipore (Bedford, MA, USA). Membranes were then blocked with 5% non-fat milk in Tris-buffer saline (TBS) 1x buffer containing (blocking solution) and incubated under gentle agitation overnight at room temperature in the presence of the primary antibodies. After that, the membranes were incubated at 37 °C for 30 min with the secondary fluorochrome-conjugated LI-COR antibody. Infrared fluorescence detection was then used for protein quantification. Membranes were scanned by the scanner Odyssey (LI-COR Biosciences, Lincoln, NE, USA) at 800 nm. Band volumes were quantified in the scanned images using ImageQuant TL v 2003 software (GE Healthcare, Uppsala, Sweden). For each antibody, the five samples of each genotype were analyzed on the same gel to suppress a technical effect. A pool constituted of all the samples was loaded on each gel, and the protein abundance for each sample was corrected with the value of this reference pool. The result for each protein is given in arbitrary units. The specificity and conditions of use of primary antibodies against candidate proteins were first defined. An antibody was considered specific when its target bands were detected at the expected molecular weight. The specific dilutions used for each antibody are reported in Table 1. The secondary fluorescent-conjugated (IRDye 800CW) antibodies were supplied by LI-COR Biosciences (Lincoln, NE, USA) and used at 1/20,000.

#### 2.3.4. Enzyme Activities Measurement

Glycolytic enzyme activities (phosphofructokinase (Pfk; EC 2.7.1.11) and lactate dehydrogenase (Ldh; EC 1.1.1.27)) and oxidative enzyme activities (isocitrate dehydrogenase (Icdh; EC 1.1.1.42)) were quantified as described in [14]. Two hundred mg of frozen muscle was thawed, ground and homogenized with a Polytron for 15 s in a 5% (*w*/*v*) solution buffer of 10 mM Trizma-Base (pH 8.0), 250 mM sucrose and 2 mM EDTA. One aliquot of homogenate was centrifuged at 6000 g for 15 min at 4 °C for the determination of Pfk, Ldh and Icdh activities. Enzyme activities (means of triplicate) were measured at 25 °C or 28 °C using an automatic spectrophotometric analyzer (UVIKON 923, Biotek-Instruments, Winooski, VT, USA). The results were expressed as units per gram of wet muscle or per μg of proteins.

### 2.4. Bioinformatic Analyses

Data mining of proteomic data was realized using the ProteINSIDE workflow [15] to analyze lists of protein or gene identifiers from ruminant species and gather biological information provided by functional annotations, putative secretion of proteins and proteins interactions networks.

### 2.5. Statistical Analysis

**One-way ANOVA.** In a first step, variance analysis was processed under SameSpots to highlight spots differentially expressed between groups (*HspB*-null mice *vs.* wild-type controls) with *p* < 0.05. After that, the differences in the identified 22 muscle protein abundances, the enzymatic activities and the abundances of the proteins revealed using Western blotting between *HspB1*-null mice (*n* = 5) and wild-types (*n* = 5) were assessed by one-way analysis of variance using individual observations; the means were compared by the *post hoc* Fisher least squares difference (LSD) test. Differences were considered to be statistically significant at a confidence level of 95% (*p* < 0.05). The results are expressed as least squares of the means ± standard error of mean (SEM).

**Correlation analysis.** The PROC CORR option of the SAS software was used to determine the Pearson’s correlation coefficients between the 22 differentially abundant proteins using Z-scores. A Z-score measures the number of standard deviations an observation is away from the mean, or average, of all observations. In our case, a Z-score represents how many the number of standard deviations of each observation is relative to the mean of the corresponding protein in each gel: Z = ((x − μ)/σ), where x is the raw value, μ is the mean of the abundance (intensity) of each protein in the corresponding group, and σ is the standard deviation of the same protein for the corresponding group. Only the significant correlations (*p* < 0.05) were considered for the construction of the correlation networks following the procedure recently described by Gagaoua *et al.* [16].

**Principal Component Analysis (PCA).** The PCA procedure is an unsupervised method which condenses the differential proteins into a set of representative, uncorrelated principle components (PCs) by means of their variance covariance structure. It was carried out using the PROC PRINCOMP from SAS, using only the 22 proteins. The PCA aimed to visually illustrate the identified differential proteins according to the studied group (*HspB*-null mice *vs.* wild-type controls).

## 3. Results

### Changes in Protein Profiles

**Figure 1:**
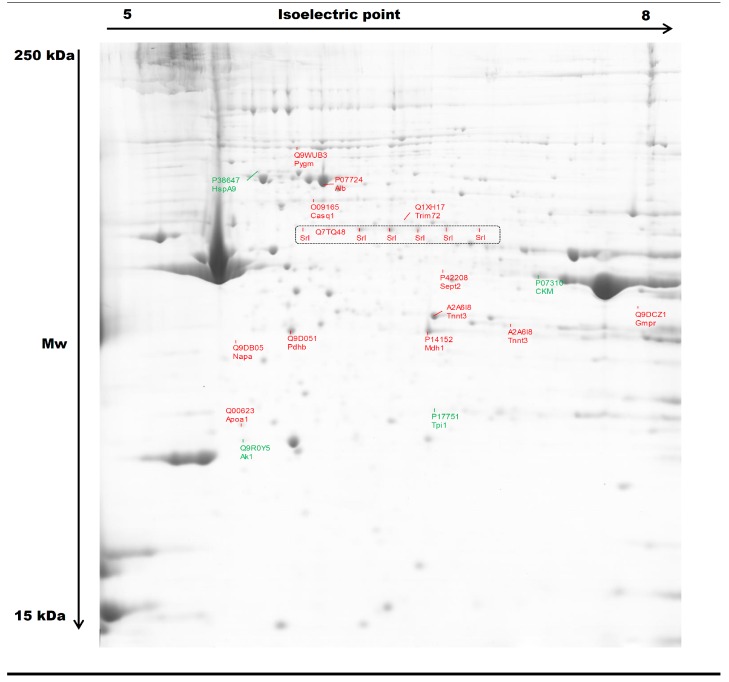
Representative 2-Dimensional gel electrophoresis (DE) map of proteins extracted from *Tibialis anterior* muscle. 2-DE was performed using a 5–8 pH gradient in the first dimension and SDS-PAGE (T = 11%, C = 2.6%) in the second; 700 μg of protein were loaded. Two-DE gels were stained with G250 Colloidal Coomassie Blue. Proteins more abundant in *HspB1* -null mice are presented in red and those less abundant are presented in green. The protein names are presented in Table 2. Mw: Molecular Weight; kDa: kilodalton.

**Figure 2:**
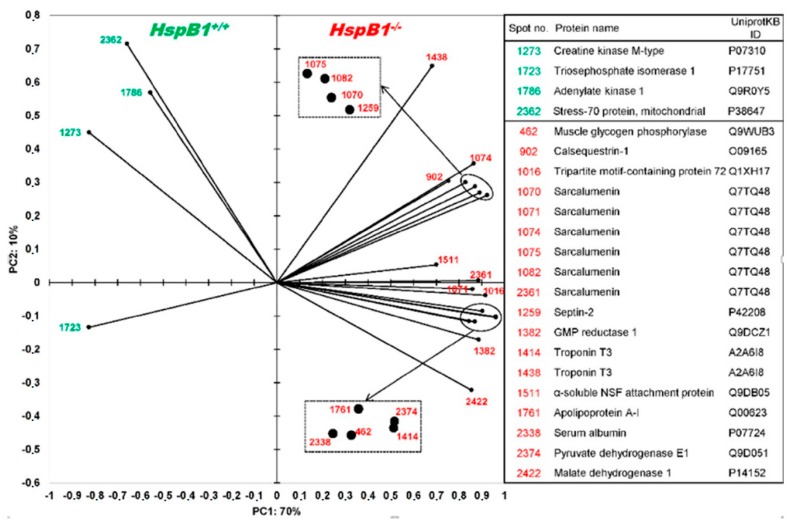
Principal component analysis (PCA) of the differential proteins according to the studied group (*HspB*-null mice *vs.* wild-type controls). The protein names are given on the right of the PCA and more details are presented in Table 2. Proteins more abundant in *HspB1*-null mice are presented in red and those less abundant are presented in green.

**Figure 3:**
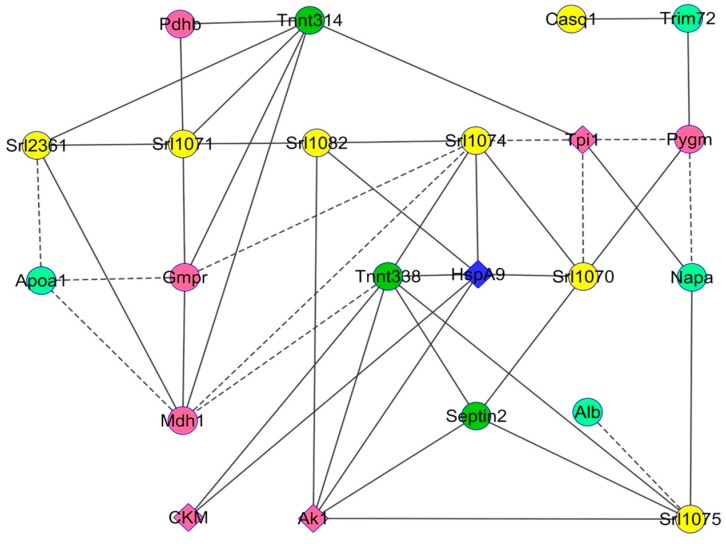
Correlation network within the two groups between the identified protein spot abundances. The Pearson’s correlation coefficients (*p* < 0.05) between the 22 differentially abundant proteins on Z-scores were computed using the Proc CORR of SAS. The solid and dash lines represent the positive and negative correlations, respectively. The circle nodes represent the 18 proteins more abundant in *HspB1*-null mice, and the lozenge nodes the four less abundant proteins. The colors of the nodes are as follow: Yellow for calcium homeostasis; green for structural proteins; blue for heat shock proteins; pink for metabolism proteins; and green watercolor for transport proteins. (For interpretation of the references to color in this figure legend, the reader is referred to the web version of this article).

**Figure A1:**
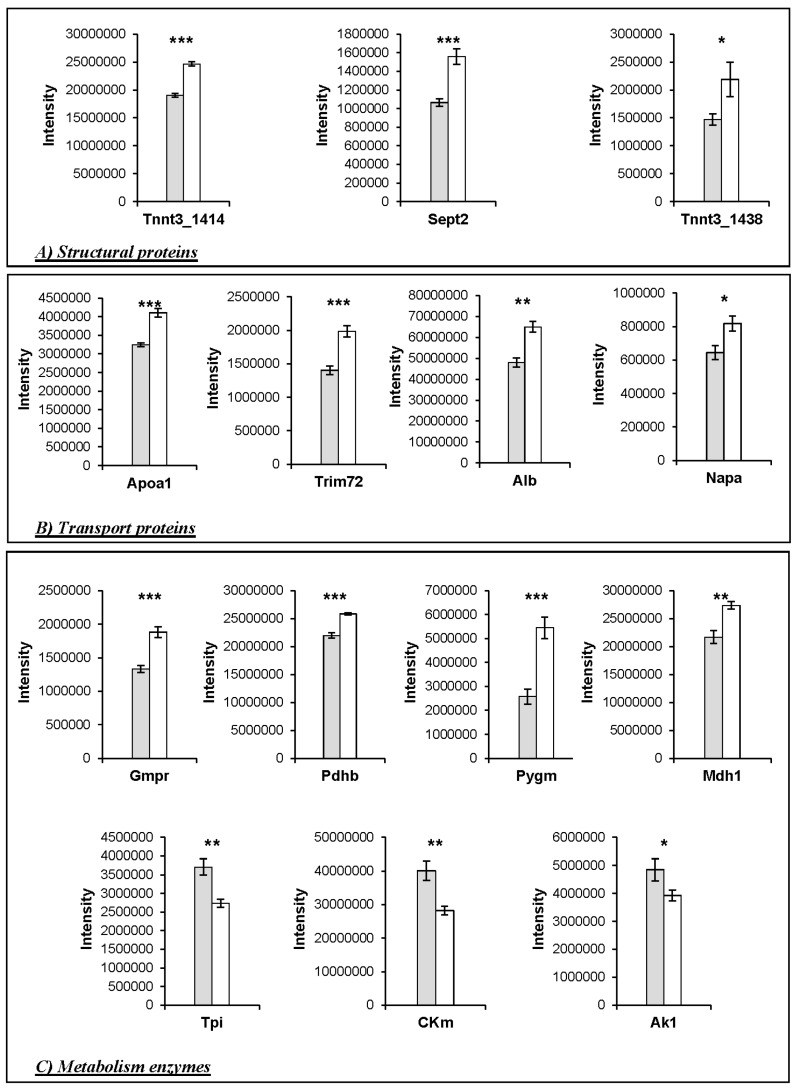

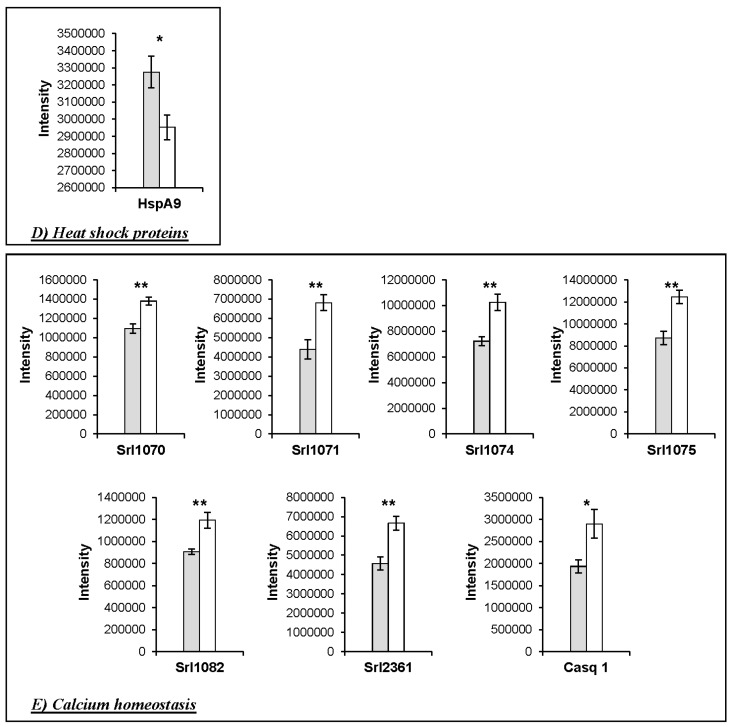
**P**rotein spots significantly differentially abundant between *HspB1*-null mice (in **white**) and wild-type controls (in **grey**). Significance: *: *p* < 0.05; **: *p* < 0.01; ***: *p* < 0.001. Intensity of 2-DE spots is expressed in arbitrary units. (**A**–**E**) indicate the biological functions in which the proteins are involved.

About 464 protein spots were detected in the reference gel from p*I* 5.5 to p*I* 7.8, and over the total mass ranges from 15 to 230 kDa (kilodalton). Among them, the comparative proteomic analysis revealed 35 spots with significant differences of abundances between *HspB1*-null mice and their controls (*p* < 0.05). Mass spectrometry allowed the identification of 16 proteins with different Uniprot ID corresponding to 22 spots (Figure 1, Table 2). Eighteen protein spots were more abundant in the *Tibialis anterior* muscle of the *HspB1*-null mice, and four proteins were less abundant (Figure 2, Table 2). Interestingly, among the spots, six isoforms of a same protein (Srl, sarcalumenin) were found differential between the two groups. All these spots were more abundant (*p* < 0.05) in *HspB1*-null mice (Figure 1 and Figure 2). The most important differences between the two groups were observed for the proteins TnnT3, Sept2, Apoa1, Trim72, Pygm, Pdhb and Gmpr. They were highly and significantly more abundant in *HspB1*-null mice compared to the controls (Figure A1). The analysis of correlations using Z-scores between the 22 protein spot abundances allowed us to construct a robust correlation network (Figure 3). The network clearly demonstrates that proteins with the higher number of interactions with other proteins were Mdh1 (six correlations) and two spots of TnnT3 (with six correlations for each). Ak1 had five positive correlations, two with Srl spots. The Srl spots showed higher numbers of correlations, and seven correlations for Srl1074. HspA9 was also correlated with six other protein spots. These numerous correlations between the differential spots identified constitute strong arguments validating the proteomic results.

**Figure 4:**
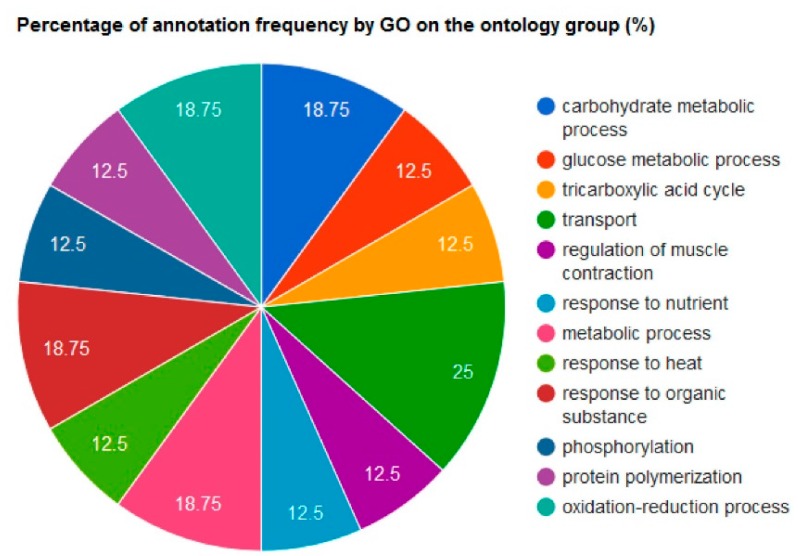
The Gene Ontology of the 16 proteins differential proteins following HspB1 invalidation. The percentage of annotation frequency by Gene ontology (GO) on the Biological Process ontology group has been retrieved by ProteINSIDE (http://www.proteinside.org/).

The list of 16 differential proteins was then submitted to ProteINSIDE [15]. A gene ontology analysis retrieved 302 GO for 16 proteins as follows: 23.2% were annotated for Cellular Component; 23% for Molecular Function; and 53.3% for Biological Process. Further data mining indicated that three proteins were mitochondrial (Mdh1, Pdhb, and HspA9) as determined by ProteINSIDE. Two proteins (Alb and Apoa1) were identified as secreted proteins based on their SignalP and TargetP scores and their GO related to secretion. Eight other proteins (Napa, Tpi1, Sept2, Pdhb, Pygm, HspA9, Ak1 and Mdh1) were also characterized by a GO related to secretion (Table 3). Interestingly, the 10 proteins predicted to be secreted were all annotated for extracellular exosome (GO:0070062). The percentage of annotation frequency by GO on the ontology group Biological Process is presented in Figure 4. The main functions represented in the dataset were related to energy metabolism with a focus on carbohydrate metabolism, muscle contraction and transport.

**Figure 5:**
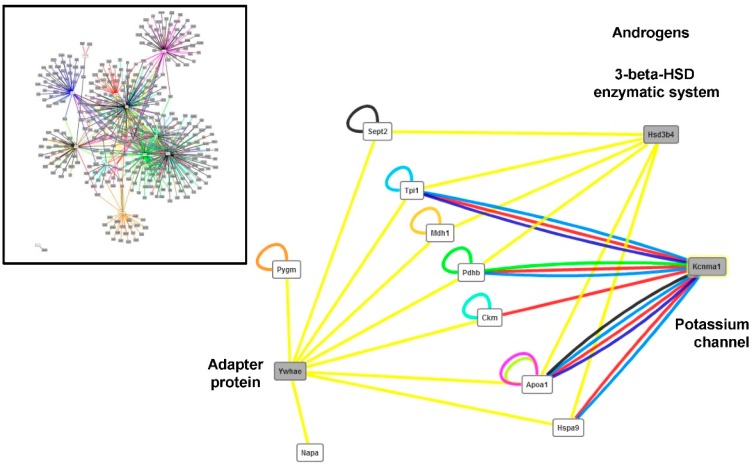
Protein-Protein interaction (PPI) outside the dataset (after enrichment by mouse proteins). Left panel: Cytoscape view of the 603 interactions detected by 415 proteins (14 from the dataset). Right panel: Network after using the filter closeness centrality (min 0.1–max 0.51) and betweeness centrality (min 2500–max 3000). The protein names are presented in Table 2.

The list of proteins was computed for direct Protein-Protein interactions (PPI) within the dataset and outside the dataset after enrichment with mouse proteins. Ten interactions were detected within the dataset. Interestingly, 603 interactions were detected outside the dataset for 415 proteins including 14 of the differential proteins. Figure 5 illustrates the PPI network before and after filtering for closeness centrality (min 0.1–max 0.51) and for betweeness centrality (min 2500–max 3000). The data indicated that three proteins (Ywhae, Kcnma1, and Hsd3b4) were highly connected to nine proteins with differential abundance in the muscle of the *HspB1*-null mouse. In particular, based on co-sedimentation experiments, the adapter protein Ywhae was predicted to be an interactor of these nine proteins, of which there are metabolic enzymes (Tpi1, Mdh1, PdhB, Ckl, Pygm), Sept2, Napa, ApoA1, and HspA9. The 3β-hydroxysteroid dehydrogenase was connected to the same proteins, except for Pygm and Napa, also based on co-sedimentation experiments. Lastly, the potassium channel activated by both membrane depolarization and the increase in cytosolic calcium was found to be an interactor of the metabolic enzymes Tpi1, Pdhb, Ckm; this was also the case for the HspA9 and ApoA1 proteins, as predicted by affinity chromatography technology, anti-tag co-immunoprecipitation, anti-bait co-immunoprecipitation, or molecular interaction experiments.

We selected a list of six proteins for validation using Western blotting according to the statistical significance of the differences in spot abundances between *HspB1*-null animals and their controls, using the available antibodies. Table 4 illustrates the Western blotting data which allowed for the validating of the lower abundance of HspA9 in *HspB1*-null mice as expected according to Table 2. The differential abundance of three proteins (Pygm, Casq1, Alb) could not be validated (Table 4).

**Figure 6:**
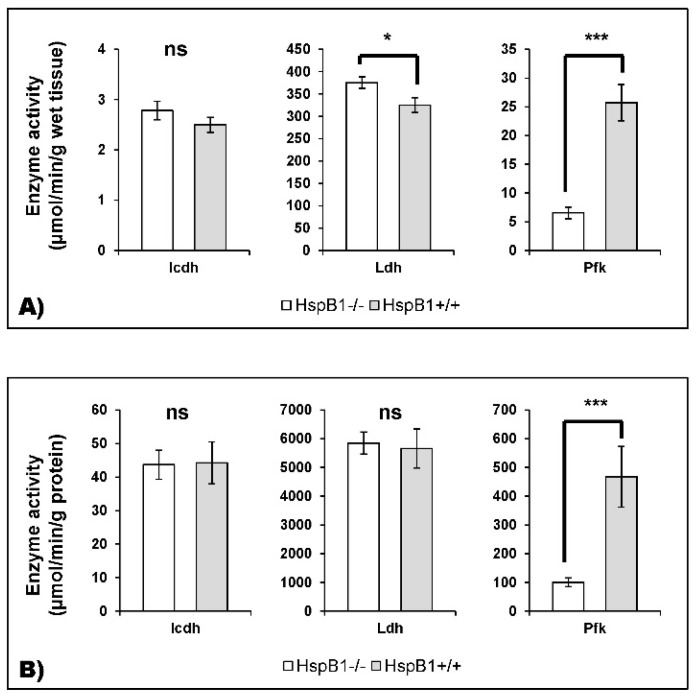
Enzymatic activity of Isocitrate dehydrogenase (Icdh, oxidative), Lactate dehydrogenase (Ldh, glycolytic) and Phosphofructokinase (Pfk, glycolytic) is measured in (**A**) μmol/min/g wet muscle tissue and (**B**) μmol/min/g protein. Histograms are in *HspB1*-null mice in white and in wild-type controls in grey. * *p* < 0.05; *** *p* < 0.001; ns: not significant.

Because significant modifications in the abundance of several enzymes of muscle energy metabolism were observed, we measured the activity of three muscle enzymes representative of oxidative (Icdh) or glycolytic (Ldh and Pfk) metabolism. The results show a highly significant difference for Pfk activity (*p* < 0.001) between the two groups. The Pfk activity was considerably lower in the *HspB1*-null mouse (Figure 6). The Ldh activity expressed in μmol/min/g of muscle was found to be slightly and significantly different (*p* < 0.05) between the two groups, and was higher in the *HspB1*-null mouse than in the wild-type. Finally, no significant difference (*p* > 0.05) was observed for Icdh activity between the two groups (Figure 6).

## 4. Discussion

In this study we have examined the proteomic signature of the *Tibialis anterior* muscle in the *HspB1*-null mouse and compared it to that of control mouse. The differential proteins between the two genotypes were mostly more abundant in the *HspB1*-null animals comparatively to the wild-type controls. The most important differences were found for the proteins of the calcium homeostasis and the energy metabolism.

### 4.1. Changes in the Abundance of Proteins Involved in Calcium Homeostasis in the HspB1-NullMouse

The proteomic analysis allowed us to detect two proteins involved in calcium homeostasis as differentially abundant in the mutant mouse, namely, sarcalumenin (Srl) with six spots, and calsequestrin (Casq1).

In skeletal muscle cells, fine regulation of Ca^2+^ storage, uptake and release is achieved through the concerted action of three major classes of Sarcoplasmic Reticulum (SR) calcium-regulatory proteins: calsequestrin, junctate and sarcalumenin (Srl) for calcium storage; SR calcium release channels such as ryanodine receptors (RyR) for calcium release; and SR Ca^2+^–ATPase (SERCA) pumps for calcium reuptake [17]. Srl are major luminal glycoproteins that codistribute with SERCA and play a role in Ca^2+^ transport and sequestration [18,19]. It maintains muscle Ca^2+^ homeostasis and is involved in the sequestration of Ca^2+^ in the non-junctional region of the SR [18]. The expression of Srl is similar in slow and fast-twitch muscle fibers. After the initiation of muscle contraction by increasing cytoplasmic Ca^2+^, Ca^2+^ is pumped back to the SR by SERCA, leading to relaxation. Srl-deficient mice were apparently normal in growth, health, and reproduction, indicating that Srl is not essential for fundamental muscle functions. Srl-deficient skeletal muscle carrying irregular SR ultrastructure retained normal force generation but showed slow relaxation phases after contractions. A weakened Ca^2+^ uptake activity was detected in the SR prepared from Srl mutant muscle, indicating that Srl contributes to Ca^2+^ buffering in the SR lumen, and also to the maintenance of Ca^2+^ pump proteins [18]. There are two Srl isoforms (160-kDa and 53-kDa) that are generated by alternative splicing [18]. In the present study, six spots of the 53-kDa isoform were found to be more abundant in the muscle of the *HspB1*-null mouse, and this overexpression was confirmed by Western blotting (Table 4). These spots could correspond to phosphorylation of Srl. In accordance with this hypothesis, Hadad *et al.* [20] revealed the phosphorylation of Srl in cardiac and skeletal muscle by Casein kinase II (CkII). They demonstrated that while the phosphorylation of the purified Srl protein by exogenously added CK II is independent of Ca^2+^, the phosphorylation of the SR membrane-associated proteins is absolutely Ca^2+^-dependent. Ca^2+^ could be involved in the transport of ATP into the SR lumen or in the protein-membrane association-dissociation processes. The phosphorylation of Srl induces modifications in the ryanodine receptor properties, and both the Ca^2+^ and the ryanodine binding affinities are modified. As there are no indications for a direct interaction between Srl and the RyR, it was suggested that this effect could be done via calsequestrin. Action potentials may elicit contractions by releasing Ca^2+^ from the SR via the RyR. This latter is modulated directly or indirectly by various ions, small molecules and proteins, including calsequestrin. In the present study, a proteomic analysis of *Tibialis anterior* muscle of both groups of mice identified an up-regulation of calsequestrin-1 (Casq1, spot 902) in the *HspB1*-null mouse [21].

Casq1 is an important regulatory of Ca^2+^. It is located in the supramolecular membrane assembly in the terminal cisternae region of muscle fibers. It represents a high-capacity, medium-affinity binding protein that is restricted to junctional SR. Casq1 plays a key role in both homeostasis and terminal cisternae structure and acts as the physiological mediator of the excitation-contraction relaxation cycle through the regulation of the ryanodine receptor complex (Ryr) [22]. Casq1 isoform is expressed in fast-twitch II fibers at increasing levels from birth to adulthood [23]. Interestingly, Casq1 knock-out does not alter contractile responses of muscle fibers but induces profound ultrastructure remodeling, reducing the content of the stored Ca^2+^ and the amplitude of Ca^2+^transient [24]. This demonstrates the crucial role of the protein for the fine muscle architecture in agreement with the electronic microscopy observations in the m. *Soleus* and m. *Tibialis anterior* of the HspB1-null mouse [7].

Ca^2+^ signaling mechanisms in skeletal muscle control a multitude of cellular processes (for review [23]). Ca^2+^ ions are well known for their implication in muscle contraction. Under resting conditions, the cytosolic-free Ca^2+^ level is higher in type I than type II fibers, and this contributes to the structural differences in the composition of fiber types. Type II fibers require higher Ca^2+^ gradients for muscle contraction; this is consistent with a higher density of RyR channels in type II fibers comparatively to type I.

Ca^2+^ release is highly regulated by smaller molecules such as ATP and Mg^2+^. A major metabolic pathway in skeletal muscle that provides a high amount of ATP generation per time is the anaerobic glycolysis [23]. Ca^2+^ ions contribute to the regulation of glycolysis as they affect the enzymatic speed of several crucial enzymes of this pathway. This is consistent with our results showing modifications of abundances of the protein involved in Ca^2+^ storage and in glycolysis.

### 4.2. Changes in the Abundance of Proteins Involved in Energy Metabolism in the HspB1-Null Mouse

Overloaded sarcoplasmic concentrations of Ca^2+^ are responsible for the activation of muscle metabolism and acceleration of lactate production in *postmortem* muscle [25]. These high Ca^2+^ concentrations have also been implicated as initiators of apoptosis via some signaling pathways in skeletal muscle [26,27]. In this study, seven proteins involved in energy metabolism were reported to be different between the two groups. Moreover, one major result of this study is the very low phosphofructokinase (Pfk) activity observed in the *HspB1*-null mouse. Pfk is the main rate-controlling enzyme of glycolysis in various tissues which catalyzes the transfer of a phosphoryl group from ATP to fructose-6-phosphate to yield ADP and fructose-1,6-bisphosphate [25]. Its activity is controlled by the concentrations of a large number of metabolites, including ATP, ADP, AMP, PEP and fructose-2,6-bisphosphate. The down-regulation of Pfk in the present study in HspB1-null mice, together with the finding that lactate accumulates in river prawn following hypoxia [26], may suggest that this protein may be a key factor in accelerating glycolysis to stimulate anaerobic ATP production in *postmortem* muscle. Three enzymes are candidates to be the major actors of this regulation because of their high negative free energy, namely, hexokinase, Pfk, and pyruvate kinase. Of the three, Pfk is considered to be the major regulatory enzyme for glycolysis in skeletal muscle. This energy barrier makes sense as pyruvate kinase catalyzes the final reaction and hexokinase is not involved in glycolysis at all when the process is begun from glycogen. Otherwise, the Pygm could catalyze the degradation of glycogen and lead to technological defects in meat (e.g., Pale, Soft, Exudative (PSE) meat) [27].

In humans, a muscular metabolic disorder known as glycogen storage disease type VII, characterized by glycogen storage disease (excess glycogen accumulation), is induced by Pfk deficiency. The symptoms are similar to deficiencies of phosphoglycerate kinase, phosphoglycerate mutase, lactate dehydrogenase, β-enolase and aldolase A. From these data, we can speculate that *HspB1*-null mice should be more fatigable and sensitive to physical exercise than their controls. This may be supported by the finding in the present study that the tripartite motif protein 72 protein (Trim72) was more abundant in the *HspB1*-null animals compared to wild-type controls. Indeed, Trim72 is abundantly expressed in striated muscle tissues and has been shown to function as a critical component of the cell membrane repair [28,29]. We also identified, as shown in the correlation network, interactions between Trim72 with Casq1 (a calcium-binding protein) and Pygm (involved in carbohydrate metabolic process). Moreover, alternative routes of anaerobic carbohydrate catabolism are less efficient for ATP production and probably do not provide enough energy to maintain aerobic consumption in the *HspB1*-null mice. This hypothesis needs to be validated.

On another hand, Ca^2+^ ions are able to modulate Pfk activity by the Ca^2+^-dependent activation of Calmodulin (CaM) which interacts with Pfk by binding on two binding sites on Pfk monomers. This generates stable Pfk dimers, which exhibit increased catalytic activity of Pfk, in part preventing allosteric inhibition of the enzyme, e.g., by ATP, citrate and lactate. This regulation contributes to an increase of Pfk activity via increased abundance of Ca^2+^. In the present study, we could speculate that the higher storage of Ca^2+^ through modifications of Casq1 and Srl abundances could result in a decrease of Pfk activity.

Two mitochondrial proteins of energy metabolism (Mdh1 and Pdhb) were found to be more abundant in the *HspB1*-null mouse. The Ca^2+^ influx into mitochondria increases the energy conversion potential, which is very important for the energetic homeostasis in contracting muscle. The activity of dehydrogenase enzymes such as Mdh1 and Pdhb are dependent on mitochondrial Ca^2+^ concentrations [23]. As a pluripotent organelle, the mitochondrion plays multiple roles in calcium homeostasis, apoptosis and physiology in *postmortem* skeletal muscle [30,31]. Under high calcium flux, the integrity of mitochondria would be destroyed, leading to the release of cytochrome C and other pro-apoptotic factors, which finally triggers apoptosis [17]. This may happen in the *HspB1*-null mouse as suggested in Cassar-Malek *et al.* [7].

Our proteomic analyses therefore provided strong evidences of the enhancement in both glycolytic and mitochondrial energy metabolism in the skeletal muscle of the *HspB1*-null mouse. Changes in mitochondrial oxidative capacity may be the reason of more abundant metabolic proteins in the mutant mouse.

It has been proposed by several authors [23] that Ca^2+^ ions influence the activity of Ca^2+^-sensitive phosphatases and kinases. This could explain the differences observed in Ak1 and Ckm abundances in *HspB1*-null mice, which could be the consequence of differences in Ca^2+^ in muscle fibers of the two genotypes. Other roles would be also assumed by these proteins. Ckm is an enzyme found in sarcolemma and in SR of muscle cells, where it is functionally involved in the calcium transport and ATPase activity [32]. Ckm can also act as an antioxidant by scavenging free radicals [33]. Antioxidants can minimize meat discoloration by decreasing lipid oxidation, thereby limiting metmyoglobin formation [34]. The higher expression of Ckm observed in wild-type mouse could possibly be associated with higher demand for energy during hypoxia, but it is not the case for HspB1-null mouse, where numerous enzymes of energy metabolism are highly abundant. Moreover, the increase in Mdh1 in *HspB1*-null mouse may therefore indicate a corresponding increase in oxidative phosphorylation capacity [30].

Moreover, a down-regulation of one protein spot (Spot 2362) identified as mitochondrial Hsp70 (mtHsp70; also known as GRP75 or HspA9) was found in the *HspB*-null mouse. This lower abundance in HspA9 in the *HspB1*-null mouse may be linked to better mitochondrial functioning with less reactive oxygen species production. This suggests that the mitochondria (perhaps more intact and efficient) are attempting to maintain function despite the eventual decrease of pyruvate flux into the TCA cycle. HspA9 is the only chaperone described to be regulated by glucose privation, Ca^2+^ homeostasis and perturbation of glycolysis [35]. This is consistent with the modifications of the abundance of this enzyme and with the correlations observed in the present study. It plays a key role in the folding of matrix-localized mitochondrial proteins and in the transport of proteins into the mitochondrion [36]. In cattle meat, HspA9 was related to pH3h *postmortem*, ultimate pH (pHu), and L* and b* color coordinates [37]. It plays a role in Ca^2+^ trafficking from the SR to the sarcoplasm, which may also explain relationship between HspA9 and the enzymes of the glycolytic pathway described by Gagaoua *et al.* [16] in cattle. These authors described correlations of this enzyme with proteins of glycolysis such as Mdh1 and Ldh-B, with structural proteins, proteolytic enzymes and anti-oxidant enzymes such as Park7, which was also modified in the *HspB1*-null mouse (Table 4).

### 4.3. Changes in the Abundance of Proteins Involved in Other Functions

The bioinformatic network revealed three proteins outside our dataset (Ywhae, Kcnma1, and Hsd3b4) which were highly connected to nine proteins of our dataset mainly protein of energy metabolism.

Among these three proteins, Kcnma1 proteins (Potassium Channel, Calcium Activated Large Conductance Subfamily M Alpha, Member 1), also called MaxiK channels, are composed of two subunits. The physical association between these two units is regulated by intracellular calcium and the activity is activated by both membrane depolarization, cytosolic Mg^2 + b^ or an increase in cytosolic Ca^2+^. GO annotations related to this gene include actin binding and calcium-activated potassium channel activity. These channels selectively transport K+ ions across biological membranes and play a key role in controlling the membrane potential in a number of systems [38]. Mice with an invalidation of the *Kcnma1* gene are characterized by cerebellar ataxia in the form of an abnormal conditioned eye-blink reflex, abnormal locomotion and a pronounced deficiency in motor coordination [39]. The interaction of this protein with a large number of proteins of our dataset seems to be logical, as we have seen above that the invalidation of *HspB1* had consequences for the abundances of several proteins involved in calcium metabolism.

Ywhae gene encodes tyrosine 3-monooxygenase/tryptophan 5-monooxygenase activation protein, 14-3-3 epsilon. The 14-3-3 proteins (phospho-serine/-threonine binding proteins) belong to a large, highly conserved family functioning as a dimer in diverse biological processes such as signal transduction, metabolism, protein trafficking, signal transduction, apoptosis, cell cycle regulation and potassium channel regulation [40]. The 14-3-3 proteins bind to a lot of target proteins, as observed in our bioinformatic network. A phosphoproteome study showed that 14-3-3 proteins could regulate glucose homeostasis in response to insulin or to energetic stress [41]. They are also known to sequester phosphorylated *Bad* in the cytoplasm in healthy cells and inhibit apoptosis. The 14-3-3 epsilon protein may also play a role in regulating microfilament stability during heat stress by maintaining their phosphorylation states [42,43]. This protein was also reported to interact with heat shock proteins [42,44,45,46].

Hsd3b4 (3beta-hydroxysteroid dehydrogenase type 4) belongs to the 3-β-HSD family. It plays a crucial role in the biosynthesis of all classes of hormonal steroids and catalyzes the conversion of dihydrotestosterone to 5alpha-androstanediol in the presence of the cofactor NADPH. In cattle, Guillemin *et al.* [47] observed a higher abundance of HspB1 in steers (castrated animals) comparatively to young bulls (non-castrated animals). According to this data, we could hypothesize that the androgen pathway could be modified in *HspB1*-null mice and that the androgen receptor (Ar) could be a target of *HspB1* invalidation. In accordance with this hypothesis, Zoubeidi *et al.* [48] showed that *HspB1 in vitro* knock-down induced Ar degradation via the proteasome-mediated pathway. In their study, *HspB1* knock-down inhibited the androgen-stimulated nuclear translocation of the Ar with subsequent suppression of the Ar-regulated gene expression. A recent report also indicates that Hsp27 is a mediator in the repression of Ar function [49]. In cattle, Guillemin *et al.* [47] also showed lower abundance of HspA8, HspA9, and Park7 in steers compared to young bulls. In accordance with the hypothesis of the modification of the androgen pathway in *HspB1*-null mice, we observed lower abundance of HspA8 and Park7 (Table 4), and of HspA8 in *HspB1*-null mice, confirming the hypothesis of relationships between these proteins. Altogether, these data suggest that Hsp27/Ar interactions could contribute to modulate the abundance of these proteins as indicated in the present study.

Altogether, our results show that the invalidation of the *HspB1* gene has direct or indirect consequences mainly on cellular homeostasis of calcium, energetic metabolism and apoptosis. These biological functions are also those that have an important role in beef sensory qualities such as tenderness.

### 4.4. Implications for Meat Quality

The results of the present study suggest relationships between Hsp27 and ATP production and, hence, the energy supply of contracting muscle regulated by the Ca^2+^-dependent enhancement of glycolytic enzyme activity and mitochondrial respiration. A relation between Hsp27 abundance and glycolytic activity has been previously described. For example, Liu *et al.* [50], in the skeletal muscle of Korean chickens, described an increase of glycolytic metabolism associated with the up-regulation of Hsp27. By proteomic analysis, these authors found also modifications of Srl abundance associated with modifications of Hsp27, and of enzymes of glycolytic metabolism, as in the present study. As a consequence, the difference in glycolytic metabolism can cause a differential rate of pH drop (due to the accumulation of lactate), which may affect directly protein degradation and meat quality traits (e.g., tenderness and water-holding capacity).

In cattle, we observed an inverse relationship between small Hsp, in particular Hsp27, and tenderness according to the type of muscle. In fast glycolytic muscle like the *Tibialis anterior*, Hsp27 was negatively correlated with tenderness, while fast or glycolytic proteins such as MyHC IIx and Ldh were positively correlated with tenderness. In a slow oxidative muscle these relationships are reversed [51]. This illustrates an opposite relationship between Hsp27 and proteins representative of fast glycolytic type in agreement with the present results showing higher abundance of some fast glycolytic proteins in the *HspB1*-null mice. In the present study, we failed to find differences in the abundances of small Hsps, namely, Hsp20 and αB-crystallin, described in the m. *Soleus* of the *HspB1*-null mouse [6]. This suggests a muscle-specific effect of the invalidation of *HspB1*. Consistently, an inverse *postmortem* evolution of Hsp27 according to muscle type has been described [52].

The modifications in Ca^2+^ homeostasis and of energy metabolism observed consequently to the invalidation of *HspB1* gene could be linked to *postmortem* modifications of muscle with crucial role on the establishment of meat quality. The *postmortem* degradation of ATP, and the increase of the activity of glycolytic enzymes are associated with pH drop [53]. These events are controlled by muscle Ca^2+^ homeostasis. Therefore, it is well documented that proteins involved in Ca^2+^ regulation are important for meat quality [54]. Aberrant calcium regulation in early *postmortem* period was reported to be associated with inferior meat quality [55]. For example, birds with PSE meat failed to respond to heat stress because of a delay in the up-regulation of the Ca^2+^-regulating genes *Ryr* and *Casq1*. It is also known that fast glycolysis and a rapid buildup of lactate in early-stage *postmortem* muscle may be a direct cause of the PSE defect [56]. Thus, insight into the metabolism pathways will help us to understand and prevent the occurrence of PSE meat. Otherwise, the presence of elevated sarcoplasmic Ca^2+^ concentrations soon after animal bleeding in PSE meat has been well documented [57]. The components of calcium channels located in the membrane of SR have been demonstrated to be correlated with the variation of calcium concentration in sarcoplasm of many tissues, including skeletal muscle [17]. For example the expression variance of SERCA can induce skeletal muscle Ca^2+^ disorders and PSE meat appearance [58]. Further, functional and morphological disorders of mitochondria were found in PSE meat [59], which were considered to induce or represent the appearance of apoptosis in skeletal muscle [60]. Another interesting link between apoptosis and muscle-to-meat conversion relies to the Ca^2+^ content of muscle cells. Earlier studies reported the acceleration of the tenderization process in meat administered with exogenous Ca^2+^ [60]. The protein 14.3.3 epsilon, for which a role in meat tenderness has never been described in the literature, was found with a higher abundance in group of *Longissimus thoracis* muscles of high tenderness in the French Blonde d’Aquitaine beef breed [61]. Consequently, a role in tenderness could be explained by its implication in the protection of cells from apoptosis.

On the other hand, in meat, it is well documented that skeletal muscle alterations induced by modification of Ca^2+^ flux in SR during ageing result in protein proteolysis involving ultra-structural modifications. α-actin is one of the myofibrillar proteins the most concerned by this proteolysis. As Hsp27 is known to protect α-actin, lower α-actin abundance in the muscle of *HspB1*-null animals could be expected. This was confirmed by Western blotting analysis in the present study.

Moreover, Pfk, whose activity was profoundly decreased in the *HspB1*-null mutants, plays a role in coupling glycolysis to many metabolic pathways, and would affect the technological traits of meat. For example, Krischek *et al.* [62] reported that Pfk activity was greater in pork with a faster pH drop.

## 5. Conclusions

In conclusion, proteomics allowed us to reveal proteins in relation to Hsp27 in the *Tibialis anterior* muscle of the mouse. The present data are complementary to those obtained in a previous analysis focused on the direct interactors of Hsp27 [6], in which we reported muscle-specific differences in the *HspB1*-null mouse. In the present study, the proteins altered after *HspB1* invalidation belonged mainly to calcium homeostasis, energy metabolism and apoptosis pathway. These functions are important for the conversion of muscle into meat. These data allow us to hypothesize a crucial role for the Hsp27 targets in meat tenderization. The associated pathways could account for the determinism of tenderness in glycolytic muscle. This will be further investigated in bovine muscles.

## Figures and Tables

**Table 1:** Suppliers and conditions for each antibody used in this study.

Protein	Protein Name	Primary Antibody Type	References	Dilution
Pygm	Glycogen phosphorylase	Polyclonal	Santa Cruz: SC-46347	1/1000
Casq1	Calsequestrin-1	Monoclonal	Santa Cruz: SC-137080	1/1000
Srl	Sarcalumenin	Monoclonal	Santa Cruz: SC-58845	1/1000
HspA9	Stress-70 protein, mitochondrial	Monoclonal	R & D Systems: MAB3584	1/200
Mdh1	Malate dehydrogenase	Monoclonal	Rockland 100-601-145	1/1000
Alb	Albumin	Polyclonal	Polycsiences: 23715-5	1/100
Park7	Parkinson disease protein 7 homolog	Polyclonal	Santa Cruz: SC-32874	1/250
HspA8	Heat shock cognate 71 kDa (kilodalton) protein	Monoclonal	Santa Cruz: SC-59572	1/250

**Table 2:** List of identified proteins differentially abundant in the m. *Tibialis anterior* between *HspB1*-null mice and their control littermates.

Spot_ID	UniprotKB ID	Protein	Protein Name	Score	ΣCoverage	Mr [kDa]	Calculated. pI	Anova (*p*)	Fold	Effect
***Structural proteins***										
1414	A2A6I8	Tnnt3	Troponin T fast	4530.73	43.51	28.3	9.36	0.0000	1.30	KO > WT
1438	A2A6I8	Tnnt3	Troponin T fast	1575.36	41.84	28.3	9.36	0.0571	1.49	KO > WT
1259	P42208	Sept2	Septin-2	1415.13	48.20	41.5	6.55	0.0006	1.46	KO > WT
***Transport proteins***										
2338	P07724	Alb	Serum albumin	7254.76	72.53	68.6	6.07	0.0011	1.35	KO > WT
1761	Q00623	ApoA1	Apolipoprotein A1	1249.41	46.59	30.6	5.73	0.0001	1.26	KO > WT
1016	Q1XH17	Trim72	Tripartite motif-containing protein 72	760.45	37.46	32.2	5.74	0.0006	1.41	KO > WT
1511	Q9DB05	Napa	α-soluble NSF attachment protein	2396.08	81.02	33.2	5.45	0.0221	1.27	KO > WT
***Heat shock proteins***										
2362	P38647	HspA9	Stress-70 protein, mitochondrial	1005.69	35.79	73.4	6.07	0.0253	1.11	WT > KO
***Metabolism***										
1786	Q9R0Y5	Ak1	Adenylate kinase isoenzyme 1	2595.48	62.37	21.5	5.81	0.0675	1.24	WT > KO
1273	P07310	Ckm	Creatine kinase M	4104.48	53.02	43.0	7.06	0.0061	1.42	WT > KO
1723	P17751	Tpi1	Triosephosphate isomerase 1	1694.75	51.51	32.2	5.74	0.0040	1.35	WT > KO
2422	P14152	Mdh1	Malate dehydrogenase	2973.62	48.50	36.5	6.58	0.0027	1.26	KO > WT
462	Q9WUB3	Pygm	Glycogen phosphorylase	5946.10	55.70	97.2	7.11	0.0008	2.12	KO > WT
2374	Q9D051	Pdhb	Pyruvate dehydrogenase E1 component subunit β	4353.89	55.71	38.9	6.87	0.0000	1.18	KO > WT
1382	Q9DCZ1	Gmpr	Guanosine 5′-monophosphate oxidoreductase 1	1127.11	59.71	37.5	7.09	0.0004	1.41	KO > WT
***Calcium homeostasis***										
902	O09165	Casq1	Calsequestrin-1	1176.41	26.17	46.3	4.12	0.027	1.50	KO > WT
1070	Q7TQ48	Srl	Isoform 2 of Sarcalumenin	1260.25	55.30	54.3	6.64	0.0017	1.26	KO > WT
1071	Q7TQ48	Srl	Isoform 2 of Sarcalumenin	4730.75	65.47	54.3	6.64	0.0051	1.55	KO > WT
1074	Q7TQ48	Srl	Isoform 2 of Sarcalumenin	1576.43	57.20	54.3	6.64	0.0033	1.42	KO > WT
1075	Q7TQ48	Srl	Isoform 2 of Sarcalumenin	4266.69	58.26	54.3	6.64	0.0026	1.43	KO > WT
1082	Q7TQ48	Srl	Isoform 2 of Sarcalumenin	886.87	51.91	54.3	6.64	0.0052	1.32	KO > WT
2361	Q7TQ48	Srl	Isoform 2 of Sarcalumenin	4731.14	60.17	54.3	6.64	0.0028	1.46	KO > WT

KO: *HspB1*-null mice; WT: wild-type mice (controls).

**Table 3:** Prediction of secreted proteins in the protein dataset as computed by ProteINSIDE.

UniprotKB ID	Gene Name	Peptide	SignalP Score	GO Related to Secretion	Number of GO	TargetP	TargetP Score
Q00623	ApoA1	noTM	0.899	GO:0072562 GO:0034361 GO:0034366 GO:0034364 GO:1903561 GO:0070062 GO:0042627	7	Signal peptide	1
P07724	Alb	noTM	0.852	GO:0005783 GO:0072562 GO:0070062 GO:0005794	4	Signal peptide	3
Q7TQ48	Srl	noTM	0.897		0	Signal peptide	1
O09165	Casq1	noTM	0.744		0	Signal peptide	3
Q9DB05	Napa	-	-	GO:0070062	1	Other secretory pathway	2
P17751	Tpi1	-	-	GO:0070062	1	Other secretory pathway	1
P42208	Sept2	-	-	GO:0070062	1	Other secretory pathway	2
Q9D051	Pdhb	-	-	GO:0070062	1	Mitochondrial	1
Q9WUB3	Pygm	-	-	GO:0070062	1	-	4
P38647	HspA9	-	-	GO:0070062	1	Mitochondrial	2
Q9R0Y5	Ak1	-	-	GO:0070062	1	Other secretory pathway	2
P14152	Mdh1	-	-	GO:0070062	1	-	-

**Table 4:** Abundance of selected proteins in the m. *Tibialis anterior* of *HspB1*-null mice *vs.* control mice.

Proteins	*HspB1*-Null^−^	WT	SEM	Significance
Pygm	61.072	64.897	4897	ns
Casq1	107.586	103.184	5015	ns
HspA9	20.946	40.914	3583	**
Mdh1	93.353	161.232	23.589	t
Srl	18.690	17.144	489	t
Alb	955.617	975.046	20.705	ns
HspA8	233.744	404.037	32.292	**
Park7	328.440	383.780	17.540	*

The abundances of the six proteins were measured by Western blotting. The protein names are presented in Table 2. Protein abundance for each sample is given in arbitrary units. The absence of Hsp27 was checked in *HspB1* null-mice *vs.* wild-type (WT) animals in a previous study [6]. t: tendency *p* < 0.1; *: *p* < 0.05; **: *p* < 0.01, ns: not significant; *HspB1*-null mice (*n* = 5); WT mice (*n* = 5); SEM: standard error of mean.

## References

[1] Verbeke W., Van Wezemael L., de Barcellos M.D., Kügler J.O., Hocquette J.-F., Ueland Ø., Grunert K.G. (2010). European beef consumers’ interest in a beef eating-quality guarantee. Appetite.

[2] Picard B., Lebret B., Cassar-Malek I., Liaubet L., Berri C., Le Bihan-Duval E., Hocquette J.F., Renand G. (2015). Recent advances in genomics for meat quality management. Meat Sci..

[3] Picard B. Relationship between Muscle Fibers, Growth Efficiency and Beef Quality. Proceedings of the 8th SIMCORTE.

[4] Picard B., Cassar-Malek I., Guillemin N., Bonnet M., Moo-Young M. (2011). Quest for novel muscle pathway biomarkers by proteomics in beef production. Comprehensive Biotechnology.

[5] Ouali A., Herrera-Mendez C.H., Coulis G., Becila S., Boudjellal A., Aubry L., Sentandreu M.A. (2006). Revisiting the conversion of muscle into meat and the underlying mechanisms. Meat Sci..

[6] Kammoun M., Picard B., Henry-Berger J., Cassar-Malek I. (2013). A network-based approach for predicting Hsp27 knock-out targets in mouse skeletal muscles. Comput. Struct. Biotechnol. J..

[7] Cassar-Malek I., Kammoun M., Astruc T., Chambon C., Barboiron C., Delavaud A., Picard B. (2015). An hspb1-null mouse to depict the contribution of Hsp27 in beef tenderness. Proceedings of the 61th International Congress of Meat Science and Technology (ICoMST).

[8] Kammoun M., Picard B., Astruc T., Blanquet V., Cassar-Malek I. (2016). The invalidation of Hspb1 gene does not impair mouse development but alters the ultrastructural phenotype of muscles. PLoS ONE.

[9] Bouley J., Chambon C., Picard B. (2004). Mapping of bovine skeletal muscle proteins using two-dimensional gel electrophoresis and mass spectrometry. Proteomics.

[10] Bradford M.M. (1976). A rapid and sensitive method for the quantitation of microgram quantities of protein utilizing the principle of protein-dye binding. Anal. Biochem..

[11] Chevalier F. (2010). Standard dyes for total protein staining in gel-based proteomic analysis. Materials.

[12] Chaze T., Meunier B., Chambon C., Jurie C., Picard B. (2008). *In vivo* proteome dynamics during early bovine myogenesis. Proteomics.

[13] Laemmli U.K. (1970). Cleavage of structural proteins during the assembly of the head of bacteriophage t4. Nature.

[14] Jurie C., Ortigues-Marty I., Picard B., Micol D., Hocquette J.F. (2006). The separate effects of the nature of diet and grazing mobility on metabolic potential of muscles from charolais steers. Livest. Sci..

[15] Kaspric N., Picard B., Reichstadt M., Tournayre J., Bonnet M. (2015). Proteinside to easily investigate proteomics data from ruminants: Application to mine proteome of adipose and muscle tissues in bovine foetuses. PLoS ONE.

[16] Gagaoua M., Claudia Terlouw E.M., Boudjellal A., Picard B. (2015). Coherent correlation networks among protein biomarkers of beef tenderness: What they reveal. J. Proteom..

[17] Berchtold M.W., Brinkmeier H., Muntener M. (2000). Calcium ion in skeletal muscle: Its crucial role for muscle function, plasticity, and disease. Physiol. Rev..

[18] Leberer E., Timms B.G., Campbell K.P., Maclennan D.H. (1990). Purification, calcium-binding properties, and ultrastructural-localization of the 53,000-dalton and 160,000 (sarcalumenin)-dalton glycoproteins of the sarcoplasmic-reticulum. J. Biol. Chem..

[19] Mahaney J.E., Weis C.P., Grisham C.M., Kutchai H. (1991). Antibodies against the 53 kDa glycoprotein inhibit the rotational dynamics of both the 53 kDa glycoprotein and the Ca^2+^-atpase in the sarcoplasmic reticulum membrane. Biochim. Biophys. Acta BBA Biomembr..

[20] Hadad N., Meyer H.E., Varsanyi M., Fleischer S., Shoshan-Barmatz V. (1999). Cardiac sarcalumenin: Phosphorylation, comparison with the skeletal muscle sarcalumenin and modulation of ryanodine receptor. J. Membr. Biol..

[21] Beard N.A., Laver D.R., Dulhunty A.F. (2004). Calsequestrin and the calcium release channel of skeletal and cardiac muscle. Prog. Biophys. Mol. Biol..

[22] Ohkura M., Furukawa K.I., Fujimori H., Kuruma A., Kawano S., Hiraoka M., Kuniyasu A., Nakayama H., Ohizumi Y. (1998). Dual regulation of the skeletal muscle ryanodine receptor by triadin and calsequestrin. Biochemistry.

[23] Gehlert S., Bloch W., Suhr F. (2015). Ca^2+^-dependent regulations and signaling in skeletal muscle: From electro-mechanical coupling to adaptation. Int. J. Mol. Sci..

[24] Tomasi M., Canato M., Paolini C., Dainese M., Reggiani C., Volpe P., Protasi F., Nori A. (2012). Calsequestrin (CASQ1) rescues function and structure of calcium release units in skeletal muscles of CASQ1-null mice. Am. J. Physiol. Cell Physiol..

[25] Carafoli E. (2002). Calcium signaling: A tale for all seasons. Proc. Natl. Acad. Sci. USA.

[26] Demaurex N. (2003). Cell biology: Apoptosis—The calcium connection. Science.

[27] Ouali A., Gagaoua M., Boudida Y., Becila S., Boudjellal A., Herrera-Mendez C.H., Sentandreu M.A. (2013). Biomarkers of meat tenderness: Present knowledge and perspectives in regards to our current understanding of the mechanisms involved. Meat Sci..

[28] McNeil P.L., Kirchhausen T. (2005). An emergency response team for membrane repair. Nat. Rev. Mol. Cell Biol..

[29] Cai C., Masumiya H., Weisleder N., Matsuda N., Nishi M., Hwang M., Ko J.-K., Lin P., Thornton A., Zhao X. (2009). Mg53 nucleates assembly of cell membrane repair machinery. Nat. Cell Biol..

[30] Contreras L., Drago I., Zampese E., Pozzan T. (2010). Mitochondria: The calcium connection. Biochim. Biophys. Acta BBA Bioenerg..

[31] Mitsui T., Endo I., Matsumoto T., Umaki Y., Akaike M. (2002). Apoptosis-related changes in skeletal muscles of patients with mitochondrial diseases. Acta Neuropathol..

[32] Rossi A.M., Eppenberger H.M., Volpe P., Cotrufo R., Wallimann T. (1990). Muscle-type MM creatine kinase is specifically bound to sarcoplasmic reticulum and can support Ca^2+^ uptake and regulate local ATP/ADP ratios. J. Biol. Chem..

[33] Lawler J.M., Barnes W.S., Wu G., Song W., Demaree S. (2002). Direct antioxidant properties of creatine. Biochem. Biophys. Res. Commun..

[34] Decker E.A., Livisay S.A., Zhou S., Decker E.A., Faustman C., Lopez-Bote C.J. (2000). Mechanisms of endogenous skeletal muscle antioxidants: Chemical and physical aspects. Antioxidants in Muscle Foods: Nutritional Strategies to Improve Quality.

[35] Mayer M.P. (2013). Hsp70 chaperone dynamics and molecular mechanism. Trends Biochem. Sci..

[36] Wiedemann N., Frazier A.E., Pfanner N. (2004). The protein import machinery of mitochondria. J. Biol. Chem..

[37] Gagaoua M., Terlouw E.M.C., Micol D., Boudjellal A., Hocquette J.-F., Picard B. (2015). Understanding early post-mortem biochemical processes underlying meat color and pH decline in the longissimus thoracis muscle of young blond d’aquitaine bulls using protein biomarkers. J. Agric. Food Chem..

[38] Turner S.C., Shieh C.-C. (2006). Medicinal chemistry of Ca^2+^-activated K^+^ channel modulators. Triggle: Voltage-Gated Ion Channels as Drug Targets O-Bk.

[39] Sausbier M., Hu H., Arntz C., Feil S., Kamm S., Adelsberger H., Sausbier U., Sailer C.A., Feil R., Hofmann F. (2004). Cerebellar ataxia and purkinje cell dysfunction caused by Ca^2+^-activated K^+^ channel deficiency. Proc. Natl. Acad. Sci. USA.

[40] Jin D.-Y., Lyu M.S., Kozak C.A., Jeang K.-T. (1996). Function of 14-3-3 proteins. Nature.

[41] Ogihara T., Isobe T., Ichimura T., Taoka M., Funaki M., Sakoda H., Onishi Y., Inukai K., Anai M., Fukushima Y. (1997). 14-3-3 protein binds to insulin receptor substrate-1, one of the binding sites of which is in the phosphotyrosine binding domain. J. Biol. Chem..

[42] Gohla A., Bokoch G.M. (2002). 14-3-3 regulates actin dynamics by stabilizing phosphorylated cofilin. Curr. Biol..

[43] Sluchanko N.N., Gusev N.B. (2010). 14-3-3 proteins and regulation of cytoskeleton. Biochem. Moscow.

[44] Chernik I.S., Seit-Nebi A.S., Marston S.B., Gusev N.B. (2006). Small heat shock protein Hsp20 (Hspb6) as a partner of 14-3-3γ. Mol. Cell. Biochem..

[45] Dreiza C.M., Komalavilas P., Furnish E.J., Flynn C.R., Sheller M.R., Smoke C.C., Lopes L.B., Brophy C.M. (2009). The small heat shock protein, hspb6, in muscle function and disease. Cell Stress Chaperones.

[46] Satoh J.-I., Onoue H., Arima K., Yamamura T. (2005). The 14-3-3 protein forms a molecular complex with heat shock protein Hsp60 and cellular prion protein. J. Neuropathol. Exp. Neurol..

[47] Guillemin N., Jurie C., Cassar-Malek I., Hocquette J., Renand G., Picard B. (2011). Variations in the abundance of 24 proteins biomarkers of beef tenderness according to muscle and animal type. Animal.

[48] Zoubeidi A., Zardan A., Beraldi E., Fazli L., Sowery R., Rennie P., Nelson C., Gleave M. (2007). Cooperative interactions between androgen receptor (AR) and heat-shock protein 27 facilitate AR transcriptional activity. Cancer Res..

[49] Hassan S., Biswas M.H., Zhang C., Du C., Balaji K.C. (2009). Heat shock protein 27 mediates repression of androgen receptor function by protein kinase d1 in prostate cancer cells. Oncogene.

[50] Liu X.D., Jayasena D.D., Jung Y., Jung S., Kang B.S., Heo K.N., Lee J.H., Jo C. (2012). Differential proteome analysis of breast and thigh muscles between korean native chickens and commercial broilers. Asian Aust. J. Anim. Sci..

[51] Picard B., Gagaoua M., Micol D., Cassar-Malek I., Hocquette J.-F., Terlouw C. (2014). Inverse relationships between biomarkers and beef tenderness according to contractile and metabolic properties of the muscle. J. Agric. Food Chem..

[52] Jia X., Hollung K., Therkildsen M., Hildrum K.I., Bendixen E. (2006). Proteome analysis of early post-mortem changes in two bovine muscle types: *M. longissimus dorsi* and *M. semitendinosus*. Proteomics.

[53] Bendall J.R. (1973). Postmortem changes in muscle. The Structure and Function of Muscle.

[54] Vignon X., Beaulaton J., Ouali A. (1989). Ultrastructural localization of calcium in post-mortem bovine muscle: A cytochemical and X-ray microanalytical study. Histochem. J..

[55] Sporer K.R.B., Zhou H.R., Linz J.E., Booren A.M., Strasburg G.M. (2012). Differential expression of calcium-regulating genes in heat-stressed turkey breast muscle is associated with meat quality. Poult. Sci..

[56] Woelfel R.L., Owens C.M., Hirschler E.M., Martinez-Dawson R., Sams A.R. (2002). The characterization and incidence of pale, soft, and exudative broiler meat in a commercial processing plant. Poult. Sci..

[57] Greaser M.L., Cassens R.G., Briskey E.J., Hoekstra W.G. (1969). Post-mortem changes in subcellular fractions from normal and pale, soft, exudative porcine muscle. 1. Calcium accumulation and adenosine triphosphatase activities. J. Food Sci..

[58] Chai J., Xiong Q., Zhang P.P., Shang Y.Y., Zheng R., Peng J., Jiang S.W. (2009). Evidence for a new allele at the serca1 locus affecting pork meat quality in part through the imbalance of Ca^2+^ homeostasis. Mol. Biol. Rep..

[59] Malila Y., Tempelman R.J., Sporer K.R.B., Ernst C.W., Velleman S.G., Reed K.M., Strasburg G.M. (2013). Differential gene expression between normal and pale, soft, and exudative turkey meat. Poult. Sci..

[60] Cao J., Yu X., Khan M.A., Shao J., Xiang Y., Zhou G. (2011). The effect of calcium chloride injection on shear force and caspase activities in bovine longissimus muscles during postmortem conditioning. Animal.

[61] Chaze T., Hocquette J.-F., Meunier B., Renand G., Jurie C., Chambon C., Journaux L., Rousset S., Denoyelle C., Lepetit J. (2012). Biological markers for meat tenderness of the three main french beef breeds using 2-DE and MS approach. Proteomics in Foods.

[62] Krischek C., Natter R., Wigger R., Wicke M. (2011). Adenine nucleotide concentrations and glycolytic enzyme activities in longissimus muscle samples of different pig genotypes collected before and after slaughter. Meat Sci..

